# Structured additive regression models with spatial correlation to estimate under-five mortality risk factors in Ethiopia

**DOI:** 10.1186/s12889-015-1602-z

**Published:** 2015-03-19

**Authors:** Dawit G Ayele, Temesgen T Zewotir, Henry G Mwambi

**Affiliations:** School of Mathematics, Statistics and Computer Science, University of KwaZulu-Natal, Private Bag X01, Pietermaritzburg, Scottsville 3209 South Africa

**Keywords:** Child mortality, Spatial model, GAMM, Household, EDHS

## Abstract

**Background:**

The risk of a child dying before reaching five years of age is highest in Sub-Saharan African countries. But Child mortality rates have shown substantial decline in Ethiopia. It is important to identify factors affecting under-five mortality.

**Methods:**

A structured additive logistic regression model which accounts the spatial correlation was adopted to estimate under-five mortality risk factors. The 2011 Ethiopian Demographic and Health Survey data was used for this study.

**Results:**

The analysis showed that the risk of under-five mortality increases as the family size approaches seven and keeps increasing. With respect to socio-economic factors, the greater the household wealth, the lower the mortality. Moreover, for older mothers, the chance of their child to dying before reaching five is diminishes.

**Conclusion:**

The model enables simultaneous modeling of possible nonlinear effects of covariates, spatial correlation and heterogeneity. Our findings are relevant because the identified risk factors can be used to provide priority areas for intervention activities by the government to combat under-five mortality in Ethiopia.

## Background

In the Sub-Saharan African countries, child mortality rate is highest (95 per 1000 live births), about 8 times higher than that in the WHO European Region (12 per 1000 live births) [1,2]. Most of the Sub-Saharan African countries, including Ethiopia, still have very high rates which are above 100 deaths per 1,000 live births [3,4]. In 2013, the rate in low income countries was 76 deaths per 1,000 live births, i.e., more than 13 times the average rate in high income countries (6 deaths per 1,000 live births). Strategies aimed at reducing these inequities across countries and saving more children’s lives by ending preventable child deaths are important priorities [5,6].

In recent years, under-five child mortality has shown a decline in Ethiopia, Malawi and Namibia, but in other African countries it has remained the same. Child survival in the first 5 years of life is influenced by different socio-economic, demographic or geographic risk factors. Studies were conducted at sub-national or national level in different African countries and identified different socio-economic and demographic factors. The other studies focused on identifying geographical variation and they recommended under-five mortality maps as useful tool for monitoring under-five mortality in different countries [7-10].

Within a country, child mortality is expected to vary greatly across risk factors and/or spatial distributions. The attempts to spatially analyze child mortality and risk factors associated with it in Ethiopia are limited due to the lack of geo-referenced data [11]. However, recent demographic and health surveys carried out in the country have had extensive coverage and collect geographical coordinates to permit the investigation of spatial variability in under-five mortality risk. Mapping of areas with high under-five mortality under investigation is crucial in an economically constrained country like Ethiopia. The map enables efficient allocation of scarce resources. A study which uses risk factors and spatial risk mapping for Ethiopian below five years child mortality is long overdue. The study reported in the article investigated socio-economic, demographic and geographic risk factors for child mortality have significant effects. To achieve this objective, a structured additive regression model was adopted and a Bayesian framework using Markov Chain Monte Carlo (MCMC) was used to analyze the 2011 Ethiopian Demographic and Health Survey (EDHS) data.

## Methods

### Study area

Ethiopia is a Sub-Saharan country and is located in the eastern part of Africa. The first Ethiopian Demographic and Health Survey (EDHS) was conducted in 2005. Since then EDHS has been conducted every five years to provide data in demographic and health risk factors in line with the country’s health and development plans. The primary objectives of the EDHS were to provide up-to-date information for planning, policy formulation, monitoring and evaluation of population and health programmes in the country. In addition to these objectives, EDHS provides critical information for use as baseline data in monitoring and evaluation of the Growth and Transformation Plan as well as various sector development policies and programmes.

### Data source

The data used for the analysis was obtained from the 2011 EDHS which was the third country-wide Demographic and Health survey conducted in Ethiopia. The survey took place over a five-month period, from 27 December 2010 through 3 June 2011 in Ethiopia. A total of 17,817 households were selected for data collection. The sampling frame used for the 2011 EDHS was the Population and Housing Census conducted by the Central Statistical Agency (CSA) in 2007 (2007 PHC). The 2011 EDHS sample was selected using a stratified, two-stage cluster design. Enumeration Areas (EA) were the sampling units for the first stage sampling. The sample included 624 EAs or clusters, 187 in urban areas and 437 in rural areas. Data was obtained from each of the eleven geographic regions in Ethiopia.

In the selected households, women and men were interviewed separately. For estimation at the national level, all data from the survey was weighted. The interviews were conducted on 9, 096 women aged 15-49 and 6,033 men aged 15-59. Thus, the 2011 EDHS sample was designed to provide estimates for the health and demographic variables of interest for Ethiopia as a whole; urban and rural area of Ethiopia comprising 11 geographical areas. The respondents were asked questions with regards to their background characteristics (age, education, media exposure, etc.), birth history and childhood mortality, knowledge and use of family planning methods, fertility preferences, antenatal, delivery, and postnatal care, breastfeeding and infant feeding practices, vaccinations and childhood illnesses, marriage and sexual activity, women’s work and husband’s background characteristics, awareness and behavior regarding AIDS and other sexually transmitted infections (STIs), adult mortality, including maternal mortality and knowledge of tuberculosis. Besides these questions, the wealth index, such as television, bicycles for each household was computed using data on the household’s ownership of selected assets and sanitation facilities, example type of drinking water source and type of toilet facility. The EDHS datasets can be obtained upon request from MEASURE DHS. Based on the 2011 EDHS, statistical report on the data and other researches were produced. The 2011 EDHS report and other published works can be accessed from the following sources [12-15].

In this study, the effects of nonlinear risk factors and the usual fixed effects of socio-economic, demographic and geographic risk factors was considered, while accounting for spatial effects. This type of Structured additive regression (STAR) model is also known as geo-additive model. The use of this model is important because of the increasing availability of disease and environmental data. The model adequately describes the variation of the disease for valid and realistic statistical inferences. For this study, we use a fully Bayesian estimation based on Markov Chain Monte Carlo (MCMC) simulations. Making inferences based on a fully Bayesian approach is preferred because functionals of the posterior can be computed without relying on large Gaussian justification. This process helps to quantify the uncertainty in the parameters [16-18].

### Statistical methods

Structured additive logistic regression model is used for this study. This method has many advantages compared to the conventional methods. This method provides a broad and generic framework for regression models incorporating continuous, discrete and mixed discrete-continuous response distributions. This method also expands the common exponential family framework. Using this method, the general guidelines for important model choice issues such as choosing an appropriate response distribution and determining a suitable predictor specification can be obtained based on quantile residuals, the deviance information criterion and proper scoring rules. Theoretically, results on the positive definiteness of the precision matrix in the proposal densities and the propriety of the posterior can be provided. Besides this, the Bayesian approach allows to borrow extensions developed for Bayesian mean regression such as multilevel structures, monotonicity constraint estimates, variable selection and regularization priors without the necessity to re-develop the complete inferential machinery [19-21].

### Model specification

Let *y*_*i*_ be the mortality status of under-five child *i* whose outcome is death, is recorded as 1, or 0 otherwise. This data follows a Bernoulli distribution

$$ {y}_i\sim Bernoulli\left({\pi}_i\right), $$

where *π*_*i*_ is the probability of the death of a child. The risk of a child to die can be associated with explanatory variables using a generalized linear model (GLM) framework with the appropriate link function. GLMs are flexible to allow for non-normal response variables [22].

With the linear predictor ${\eta}_i= log\left(\frac{\pi_i}{1-{\pi}_i}\ \right) = {w}_i^{\hbox{'}}\ {\alpha}_i$ where ${w}_i^{\hbox{'}}\kern0.5em =\kern0.5em {\left({w}_{i1},\ .\ .\ ., {w}_{ip}\right)}^{\hbox{'}}$ the GLM can be specified. Therefore, the ordinary regression can be specified as follows:

1 $$ {\eta}_i= log\left(\frac{\pi_i}{1-{\pi}_i}\right)={\beta}_0 + {w}_i^{\hbox{'}}\ {\alpha}_i $$

where *β*_0_ is the intercept, ${w}_i^{\hbox{'}}$ is a vector of covariates and *α*_*i*_ is a vector of regression coefficients. However, the standard GLM has some disadvantages, i.e., GLM assume independent (or at least uncorrelated) observations. But, this assumption is not always satisfied, sometimes observations exhibit spatial and/or temporal dependence. This variability has to be incorporated in the model. The linear predictor modified by taking into account the spatial autocorrelation, can be given as follows

2 $$ {\eta}_i={\alpha}_0 + {w}_i^{\hbox{'}}\ {\alpha}_i + {\displaystyle \sum_{k=1}^q}{f}_k\left({x}_{ik}\right) + {\varnothing}_i + {v}_i $$

where α_0_ is the intercept, α_1_ is the parameter corresponding to the categorical fixed variables ${w}_i^{\hbox{'}} = {\left({w}_{i1},\ .\ .\ ., {w}_{ip}\right)}^{\hbox{'}}$ and *f*_*k*_ is an appropriate smoothing function of continuous covariates, *x*_*ik*_. To capture the unobserved spatial heterogeneity and over dispersion at each location, spatially unstructured random effects have been used. Equation 2 gives a class of models known as structured additive regression (STAR) models. Generalized additive models (GAM) [23], generalized additive mixed models (GAMMs) [24] and geo-additive models [16] are special cases of the STAR models. All of these models make use of smooth functions to model covariate effects on the response variable. These models are increasingly being applied to model health impacts and outcomes such as spatial variation of infectious disease [25-31].

### Prior distributions for covariates

For implementation of this model, a Bayesian approach which needs prior assumptions has to be used. In Bayesian analysis, all the regression coefficients and the smooth functions *f*_*i*_ are considered as random variables and are assigned prior distributions. The question is how to select the priors. The concept involved in this selection can be reduced to concepts which are more familiar and more closely related to prior experience. If this is accomplished, it is possible to set values which can be translated into prior distribution. Therefore, for the coefficients *α*, an independent diffuse which appropriate choice to constant can be assumed. This is used due to the absence of any prior knowledge. Therefore, the possible alternative choice is a week informative multivariate Gaussian distribution.

For the functions *f*_*j*_(*x*), *j* = 1, . . . , *p*, the Bayesian P-splines has been used [20,21]. This approach assumes that an unknown smooth function *f*_*i*_ of a covariate *x*_*j*_ can be approximated by a polynomial spline of degree *l* defined on a set of equally spaced knots ${x}_j^{min}={\zeta}_{j,0}<{\zeta}_{j,1} < .\ .\ . < {\zeta}_{j,s-1}<{\zeta}_{j,s}={x}_j^{max}$ within the domain of *x*_*j*_. Such a spline can be written in terms of a linear combination of *d* = *s* + *l* basis functions *B*_*m*_, i.e.

3 $$ {f}_j\left({x}_j\right) = {\displaystyle \sum_{m=1}^d}\kern0.5em {\xi}_{j,m}.\ {B}_m\left({x}_j\right). $$

The functions *B*_*m*_ are only positive within an area spanned by *1 + 2* knots. This leads to the B-splines form a local basis. This property is used for the construction of the smoothness penalty for P-splines. The estimation of the vector of unknown regression coefficients from the data helps to be reduced to the estimation of *f*_*i*_ (*x*_*j*_). The important factor for this procedure is the choice of appropriate number of knots. It is important to choose large number of equally spaced knots. This is suggested by Eilers and Marx [20]. Hence, for Bayesian approach, penalized splines are introduced by replacing the difference penalties with their stochastic analogues. These analogues are first or second order random walk priors for the regression coefficients. A first order random walk prior for equidistant knots is given by:

4 $$ {\xi}_{j,m}=\kern0.5em {\xi}_{j,m-1} + {u}_{j,m},\ m=2,\ .\ .\ ., d, $$

and a second order random walk for equidistant knots by

5 $$ {\xi}_{j,m}=2{\xi}_{j,m-1}-{\xi}_{j,m-2}+{u}_{j,m},\ m=3,\ .\ .\ ., d, $$

where ${u_{j,m}}^{\sim }N\left({\xi}_{j,m},{\tau}_j^2\right)$ are Gaussian errors. Diffuse priors *ξ*_*j*,1_ ∝ *const*, or *ξ*_*j*,1_ and *ξ*_*j*,2_ ∝ *const*, are chosen as initial values, respectively. The joint distribution of the regression parameters *ξ*_*j*,*m*_ for a first order random walk is defined as:

6 $$ {\xi}_{j,m}\Big|{\xi}_{j,m-1}\kern0.75em \sim N\left({\xi}_{j,m},{\tau}_j^2\right) $$

and a second order random walk is defined as:

7 $$ {\xi}_{j,m}\Big|{\xi}_{j,m-1},{\xi_{j,m-2}}^{\sim }N\left(2{\xi}_{j,m-1} - {\xi}_{j,m-2},{\tau}_j^2\right) $$

From equations 4, 5, 6 and 7, it can be seen that the first order random walk induces a constant trend for the conditional expectation of *ξ*_*j*,*m*_ given *ξ*_*j*,*m* − 1_ For the second order random walk, the results shows linear trend depending on the two previous values *ξ*_*j*,*m* − 1_ and *ξ*_*j*,*m* − 2_. Therefore, to compute the joint distribution of the regression parameters, the product of the conditional densities defined by the random walk priors is used. The general form of the prior for *ξ*_*j*_ is a multivariate Gaussian distribution with density:

$$ p\left({\xi}_j\Big|{\tau}_j^2\right) \propto exp\left(-\frac{\xi_j^{\hbox{'}}{K_j}_j}{2{\tau}_j^2}\right), $$

where the precision matrix *K*_*j*_ is penalty matrix (shrinks parameters towards zero). This value penalizes too abrupt jumps between neighboring parameters, i.e., *k*_*j*_ = *rank* (*K*_*j*_) < dim(*ξ*_*j*_) = *d*_*j*_. This value follows the prior for ${\xi}_j\Big|{\tau}_j^2$ and partially improper with Gaussian prior ${\xi}_j\Big|{\tau}_j^2 \propto N\left(0;{\tau}_j^2{K}_j^{-}\right)$. Here, ${K}_j^{-}$ is a generalized inverse of *K*_*j*_. The variance parameter ${\tau}_j^2$ controls the tradeoff between flexibility and smoothness. Therefore, large variance corresponds with a rough estimated function, and vice versa.

### Spatial components

For the spatial component, the nearest neighbor Gaussian Markov random field model is used. This model is common in spatial statistics to express prior knowledge of the spatial effects. Suppose *s ϵ* {1, . . . , *S*} represent the locations of connected locations, then the locally dependent prior probability spatial structure can be specified as:

$$ {f}_{str}(s)\Big|{f}_{str}\left({s}^{\hbox{'}}\right),s\ne {s}^{\hbox{'}},{\tau}_{str}^{2\kern1em \sim }N\left(\frac{1}{N_s}{\displaystyle \sum_{s\epsilon {\theta}_s}}\kern0.5em {f}_{str}(s),\frac{\tau_{str}^2}{N_s}\right) $$

where *N*_*s*_ is the number of adjacent spatial units and *s∈θ*_*s*_ denotes that spatial unit *s’* is a neighbor of spatial unit *s*. To ensure equal number of neighbors for each locations, a neighborhood structure can be selected based on the *k*^*th*^ nearest neighbor method. Here, *k* represents the number of neighbors. Similar to the continuous functions *f*_*j*_, the tradeoff between flexibility and smoothness is controlled by the variance parameter ${\tau}_{str}^2$. For the unstructured spatial effects, parameters *f*_*unstr*_(*s*) are assumed to be *i.i.d.* Gaussian:

$$ {f}_{unstr}(s)\Big|{\tau}_{unstr}^{2\sim }N\left(0,{\tau}_{unstr}^2\right). $$

Therefore, parameters ${\tau}_j^2,\ j=1,\ .\ .\ .,\ p,\ str,\  unstru$ are considered unknown. Therefore, in the second stage, highly dispersed inverse Gamma distributions $p\left({\tau}_j^2\right)\sim IG\left({a}_j,{b}_j\right)$ with known hyper-parameters *α*_*j*_ and *b*_*j*_ are assigned. Therefore, corresponding probability density function is expressed as:

$$ p\left({\tau}_j^2\right) \propto {\left({\tau}_j^2\right)}^{-{a}_j-1}\  exp\left(\frac{b_j}{\tau_j^2}\right). $$

The model was implemented by running the MCMC algorithm. The MCMC algorithm was made using 10,000 iterations with a burn in of 1000 and 50 thinning parameters. The chosen models were run several times. This is done to ensure the choice of priors did not influence the results. This was done by running the chosen model several times by changing the prior parameters in estimates. Fully Bayesian analyses have been carried out with BayesX, software for Bayesian inference based on MCMC techniques. For the analysis, model diagnosis was performed based on MCMC post-estimation diagnosis, besides the implemented trace and autocorrelation plots, samples of the parameters were also extracted using function sample.

### Ethical clearance

Ethical clearance for the survey was provided by the Ethiopian Health and Nutrition Research Institute (EHNRI) Review Board, the National Research Ethics Review Committee (NRERC) at the Ministry of Science and Technology, the Institutional Review Board of International Coach Federation (ICF) International, and the Centers for Disease Control and Prevention (CDC).

## Results

Before performing the GAMM analysis, bivariate tests were carried out. This was done to determine which variables to include in the model. The cross tabulation, as an initial descriptive analysis method, was performed using chi-square tests to investigate the relationship between the outcome of child mortality and several categorical socio-economic, demographic and geographic variables at the 5% level of significant.

**Figure 1:**
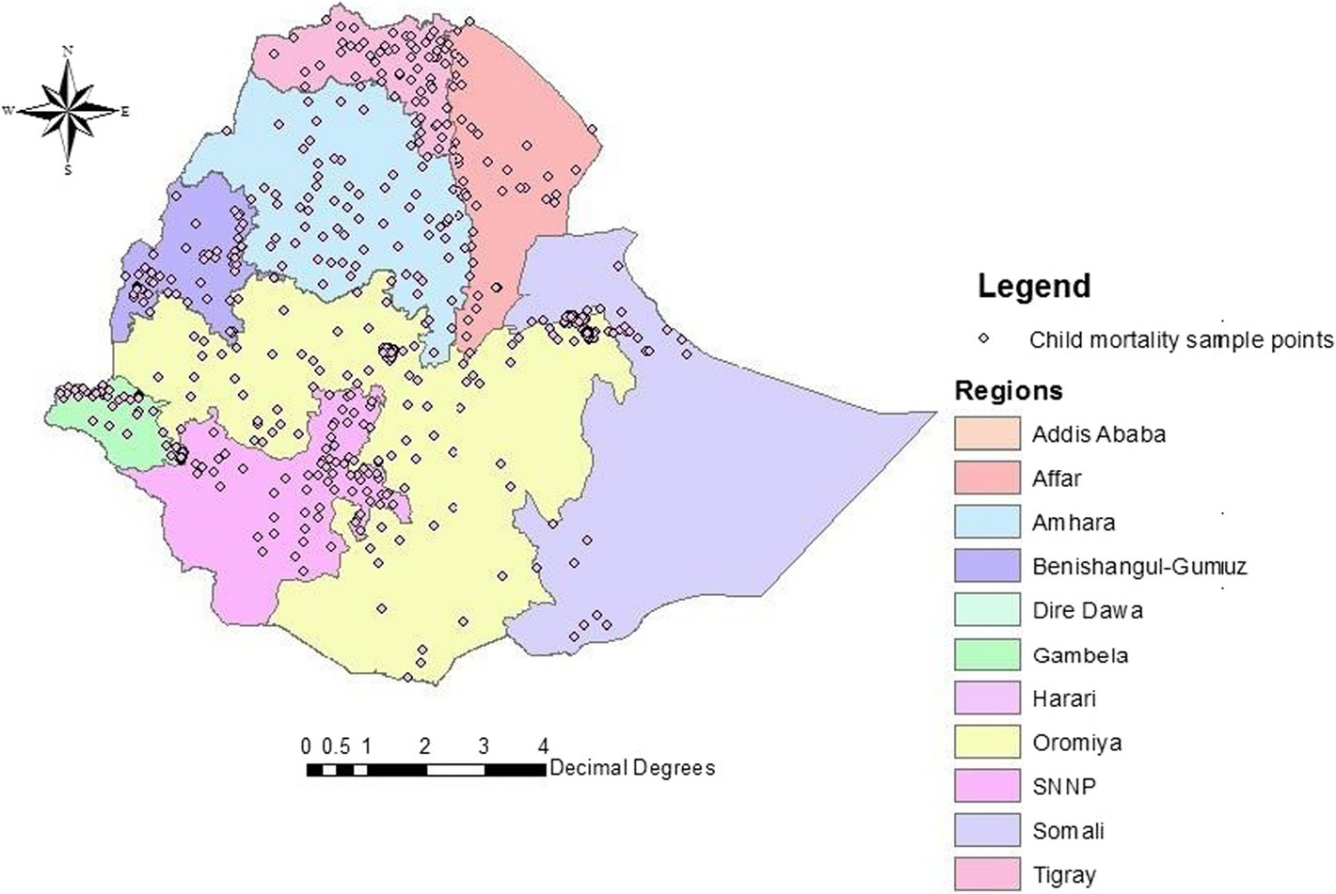
**Observed mortality risk of children under five years of age for 624 EAs across Ethiopia.**

Figure 1 shows child mortality per sampled EA across all regions of Ethiopia. It clearly demonstrates that the Tigray, Benshangul-Gumuz and SNNP regions registered higher child mortality rate than the other regions during the survey period. However, the Amhara and Oromia regions registered the lowest mortality risk.

### Bivariate association between child mortality and risk factors

Table 1 shows the association between child mortality and selected socio-economic, demographic and geographic categorical variables. At the 5% level, a statistically significant association among geographic factors; region, place of residence and child mortality status was found (p-value < 0:0001). Among demographic variables and child mortality, significant association was observed, i.e., between religion, current marital status, and child mortality status. Similarly, significant association was found between whether a child is a twin or not and child mortality status. Furthermore, the time it takes to get water, source of drinking water, type of cooking fuel, type of toilet facility, main floor material, main roof material, main wall material, wealth index show a statistically significant association with child mortality. However, the respondent’s current pregnant status did not show a statistically significant association with child mortality risk (p-value = 0.057).

**Table 1:** **Association between child mortality and socio-economic, demographic and geographic variables**

**Variables**		**Child is alive**		**P-value**
		**No (%)**	**Yes (%)**	
Region	Tigray	355 (13.26%)	2323 (86.74%)	.000
	Affar	343 (17.45%)	1623 (82.55%)	
	Amhara	666 (16.17%)	3452 (83.83%)	
	Oromiya	620 (14.36%)	3698 (85.64%)	
	Somali	232 (14.05%)	1419 (85.95%)	
	Benishangul-Gumuz	496 (20.09%)	1972 (79.90%)	
	SNNP	631 (15.36%)	3476 (84.64%)	
	Gambela	236 (18.32%)	1052 (81.68%)	
	Harari	176 (11.49%)	1356 (88.51%)	
	Addis Ababa	45 (5.46%)	779 (94.54%)	
	Dire Dawa	208 (14.65%)	1212 (85.35%)	
Type of place of residence	Urban	375 (9.895%)	3415 (90.11%)	.000
	Rural	3633 (16.09%)	18947 (83.91%)	
Religion	Orthodox	1336 (14.34%)	7982 (85.66%)	.000
	Catholic	46 (14.47%)	272 (85.53%)	
	Protestant	734 (14.53%)	4319 (85.47%)	
	Muslim	1787 (15.94%)	9423 (84.06%)	
	Traditional	40 (22.10%)	141 (77.90%)	
	Other	63 (23.08%)	210 (76.92%)	
Current marital status	Married	3862 (15.35%)	21305 (84.66%)	.002
	Living with partner	146 (12.14%)	1057 (87.86%)	
Currently pregnant	No or unsure	3550 (15.05%)	20031 (84.95%)	.057
	Yes	458 (16.42%)	2331 (83.58%)	
Sex of child	Male	2244 (16.55%)	11313 (83.45%)	.000
	Female	1764 (13.77%)	11049 (86.24%)	
Child is twin	Single birth	3763 (14.61%)	21991 (85.39%)	.000
	1st of multiple	122 (39.74%)	185 (60.26%)	
	2nd of multiple	122 (39.74%)	185 (60.26%)	
	3rd of multiple	1 (50%)	1 (50%)	
Time to get water	On the premises	186 (8.51%)	1999 (91.49%)	.000
	1 - 30 min	2132 (16.279%)	10964 (83.72%)	
	31 - 60 min	826 (15.14%)	4631 (84.86%)	
	>61 min	838 (15.48%)	4575 (84.52%)	
Source of drinking water	Tap water	805 (12.08%)	5859 (87.92%)	.000
	Protected water	1113 (16.01%)	5840 (83.99%)	
	Unprotected water	2077 (16.48%)	10525 (83.52%)	
Type of cooking fuel	Electricity/gas	29 (5.49%)	499 (94.51%)	.000
	Coal, charcoal/wood	3638 (15.45%)	19906 (84.55%)	
	Straw/animal gung	329 (15.37%)	1812 (84.63%)	
	No food cooked in the house	12 (7.64%)	145 (92.36%)	
Type of toilet facility	No toilet facility	1994 (16.28%)	10252 (83.72%)	.000
	Toilet with flush/pit latrine	1851 (14.36%)	11042 (85.64%)	
	Other toilet type	144 (13.52%)	921 (86.48%)	
Main floor material	Earth/sand/dung	3716 (16.01%)	19495 (83.99%)	.000
	Wood	51 (16.09%)	266 (83.92%)	
	Cement	230 (8.53%)	2466 (91.47%)	
Main roof material	Thatch/leaf/mud	2206 (16.80%)	10925 (83.20%)	.000
	Mat/plastic sheet/wood	248 (16.78%)	1230 (83.22%)	
	Corrugated iron/metal	1394 (13.19%)	9172 (86.81%)	
	Asbestos/cement/concrete	150 (14.15%)	910 (85.85%)	
Main wall material	Cane/trunk/bamboo	3742 (15.54%)	20337 (84.46%)	.000
	Cement/bricks	114 (8.32%)	1255 (91.67%)	
	Wood planks/shingles	142 (18.16%)	640 (81.84%)	
Wealth index	Poorest	2065 (16.7%)	10294 (83.30%)	.000
	Middle	786 (16.11%)	4093 (83.89%)	
	Richest	1157 (12.70%)	7975 (87.30%)	

### Effect of categorical variables on child mortality

Odds ratios from the best fitting model show a relationship between the socio-economic, demographic and geographic categorical variables. The risk of child mortality is shown in Tables 2 and 3. Respondents who travel greater than 60 minutes to fetch water had 9.6% higher odds of their child dying before reaching five than those who use water from other premises (adjusted OR = 1.096, CI: 1.013, 1.199). Similarly, those who travel 30-60 minutes to fetch water had 7.6% higher odds for their child to die before that age than is the case with those using water from premises (adjusted OR = 1.076, CI: 1.030, 1.086). Persons who used charcoal/wood had 2.1% higher odds of having a child who would die before reaching five years (adjusted OR = 1.021, CI: 1.001, 1.042) compared to those respondents who use straw/animal dung.

**Table 2:** **Parameter estimates for socio-economic variables**

**Parameter**	**Estimate**	**OR**	CI*	
			**Lower**	**Upper**
Intercept	−1.045	0.352	0.353	2.850
Distance to fetch water (Ref. On the premises)				
1 - 30 min	0.097	1.102	0.908	1.112
31 - 60 min	0.073	1.076	1.030	1.086
Greater than 61 min	0.091	1.096	1.013	1.199
Source of drinking water (Ref. Unprotected water)				
Protected water	0.013	1.013	0.988	1.017
Tap water	−0.061	0.941	0.841	0.986
Cooking fuel (Ref. straw/animal gung)				
Coal, charcoal/wood	0.021	1.021	1.001	1.042
Electricity/gas	−0.084	0.920	0.811	0.989
No food cooked in the house	−0.348	0.706	0.709	1.423
Toilet Facility (Ref. Toilet with flush/pit latrine)				
No toilet facility	0.036	1.037	1.005	1.157
Other toilet type	−0.052	0.969	0.850	0.998
Floor material (Ref. wood)				
Cement	0.247	1.281	1.072	1.392
Earth/sand/dung	0.296	1.345	1.044	1.466
Roof material (Ref. Thatch/leaf/mud				
Asbestos/cement/concrete	−0.049	0.952	0.943	1.051
Corrugated iron/metal	−0.004	0.996	0.886	0.998
Mat/plastic sheet/wood	−0.002	0.998	0.979	0.998
Wall material (Ref. Wood planks/shingles)				
Cane/trunk/bamboo	−0.210	0.811	0.801	0.934
Cement/bricks	−0.215	0.806	0.070	1.242
Smoking (Ref. yes)				
No	−0.215	0.807	0.601	0.987

* CI - Credible Interval.

**Table 3:** **Parameter estimates for demographic and geographic variables**

**Parameter**	**Estimate**	**OR**	**CI**	
			**Lower**	**Upper**
Region (Ref. Tigray)				
Addis Ababa	−0.210	0.811	0.809	1.235
Affar	−0.111	0.895	0.896	1.118
Amhara	0.007	1.007	1.002	1.018
Benishangul-Gumuz	0.167	1.182	1.046	1.189
Dire Dawa	0.049	1.051	1.014	1.151
Gambela	0.377	1.458	1.006	1.459
Harari	−0.246	0.789	0.782	0.985
Oromiya	0.008	1.008	0.993	1.008
SNNP	0.072	1.075	0.931	1.076
Somali	−0.040	0.961	0.962	1.041
Place of residence (Ref. Urban)				
Rural	0.090	1.094	1.014	1.099
Religion (Ref. Traditional)				
Catholic	−0.388	0.679	0.680	1.476
Muslim	−0.052	0.949	0.951	1.055
Orthodox	−0.178	0.837	0.828	1.196
Protestant	−0.180	0.835	0.816	1.199
Other	0.219	1.245	1.044	1.257
Husband’s education (Ref. Secondary)				
Don’t know	−0.154	0.858	0.849	1.168
Higher	−0.237	0.789	0.770	1.269
No education	−0.006	0.994	0.995	1.006
Primary	−0.081	0.922	0.913	1.085
Respondent currently working (Ref. Yes)				
No	−0.044	0.957	0.837	0.995
Currently pregnant (Ref. Yes)				
No/don’t know	−0.223	0.800	0.787	0.959
Sex of a child (Ref. male)				
Female	−0.239	0.787	0.747	0.971

On the other hand, respondents who uses electricity had 8% lower odds of having a child dying before five (adjusted OR = 0.920, CI: 0.811, 0.989) compared those using straw/animal dung. The interviewed who use tap water for drinking, had 5.9% lower chance of an under-five child dying compared to those who use unprotected water. Respondents using toilet with flush/pit latrine had a 3.1% lower odds of child mortality than respondents who use other toilet types (adjusted OR = 0.969, CI: 0.850, 0.998). But, no toilet facility users in the study had 3.7% higher odds of child mortality than those using other toilet types (adjusted OR = 1.037, CI: 1.005, 1.157). People surveyed in this study who live in houses with concrete floor (28%) and earth/dung/sand floors (34.5%) had higher odds of child mortality than those living in wooden floor houses (adjusted OR = 1.281, CI: 1.072, 1.392 and OR = 1.345, CI: 1.044, 1.466 respectively). Those surveyed who live in houses with corrugated iron (0.4%) and mat/plastic sheet/wood (0.2%) roof had lower chance of child mortality than respondents who live with thatched/leaf/mud roof houses (adjusted OR = 0.996, CI: 0.886, 0.998 and OR = 0.998, CI: 0.979, 0.998 respectively). The odds of child mortality steadily dropped as wealth increased. Children from medium income households were found to have 30.9% lower mortality chances than those from poor households (adjusted OR = 0.691, CI: 0. 421, 0.997). The odds further dropped with children in richest households having 75.7% lower child mortality likelihood (adjusted OR = 0.243, CI: 0.114, 0.372). Children from the central region had 48% lower odds (adjusted OR = 1.48, CI: 1.012, 2.421) than those from the poor households.

Odds ratios from the best fitting model showing the relationship between demographic and geographic categorical variables and the risk of child mortality are shown in Table 3. The table reveals that except for religion and husband’s education, the rest of the demographic and geographic variables had significant effect on child mortality in Ethiopia. Children who live in Amhara region had 0.07% higher odds of child mortality than those living in Tigray region (adjusted OR = 1.007, CI: 1.002, 1.018). Followed by Dire Dawa (adjusted OR = 1.051, CI: 1.014, 1.151), Gambella (adjusted OR = 1.458, CI: 1.006, 1.459) and Benishangul-Gumuz (adjusted OR = 1.182, CI: 1.046, 1.189) regions. In contrast, children who live in Harari region had 21% lower odds of having high mortality (adjusted OR = 0.789, CI: 0.782, 0.985) compared to child who live in Tigray region. On the other hand, children from rural areas had 9.4% higher odds of child mortality than their urban counterparts (adjusted OR = 1.094, CI: 1.014, 1.099). Respondents who are currently working had 4.3% lower odds of child mortality before the age of five than those who are not working (adjusted OR = 0.837, CI: 0.995, 1.045). Persons who are not pregnant or do not know about it had 20% lower odds (adjusted OR = 0.800, CI: 0.787, 0.959) than pregnant respondents. Similarly, the odds of a female child reaching age five is lower (21.3%) than that of a male child (adjusted OR = 0.787, CI: 0.747, 0.971).

### Effect of continuous covariates on child mortality in Ethiopia

**Figure 2:**
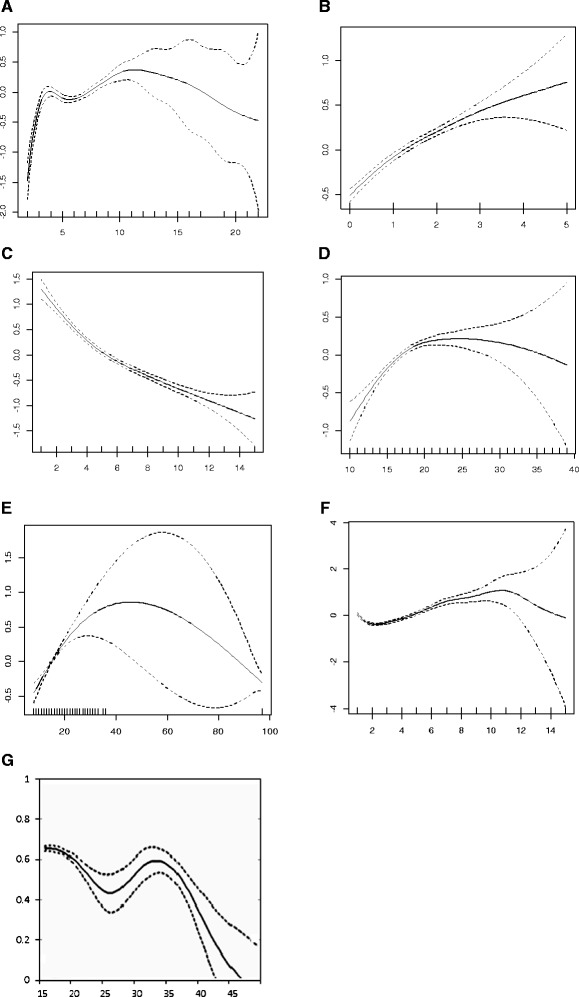
**Smoothing components for child mortality with A) Number of household members, B) Number of children 5 and under in household, C) Total children ever born, D) Age of respondent at 1st birth, E) Age at first sex, F) Birth order number, G) Age of respondent.**

In addition to categorical effects, there were continuous effects which were handled non-parametrically to the model. These effects are number of household members, number of children at age five and under in a household, age of respondent at 1st birth, age at first sex, total children ever born, age of respondent and birth order number. The result shows that age of respondent at 1^st^ birth, age at first sex, total children ever born, birth order number and age of respondent had non-linear significant effect on child mortality in Ethiopia. The possible nonlinear effects of the continuous covariates after accounting for other variables are presented in Figure 2 together with 95% credible intervals. In the figure, the dashed lines represent the 95% credible intervals. The figures suggest that age of respondents, number of household members, number of children at age five and under in a household, age of respondent at 1st birth, age at first sex, total children ever born and birth order number effects all depart dramatically from linearity. In each panel, the smooth line is the estimated trend from a generalized additive mixed model for the model with spherical Gaussian covariance structure. Figure 2A shows the estimated smooth function of family size and their 95% confidence interval. The y-axis represents the effect of the age term (posterior mean).

Furthermore, the figure suggests that for a family size of three to five, the risk of child mortality remains relatively constant. However, beginning from above seven up to eleven mortality but then starts to declining beyond twelve. Figure 2B shows an increase with the risk of child mortality as the number of children age five and under increases in the household. Figure 2C demonstrates a steady drop in child mortality with increasing total children ever born. The lowest child morality is observed at total children above nine children. On the other hand, age of respondent at first birth in Figure 2D, illustrates an association with the risk of child mortality in Ethiopia. The figure further suggested that the risk of a child to be dead before age five increases up to age 18 then starts to decrease. Similar to age of respondents at first birth, the risk of a child to be dead before age five increases up to age 20 then starts to decrease (Figure 2E). According to Figure 2F, risk increases from birth order of a child between three to ten. Child mortality risk then decreases slightly as birth order of a child increases. The other significant result was the age of respondents. The result suggests strong significant increasing nonlinear relationship between age of respondents and child mortality in Ethiopia (Figure 2G). In general, the figures suggested nonlinear relationship with child mortality in Ethiopia.

### Child mortality risk map

**Figure 3:**
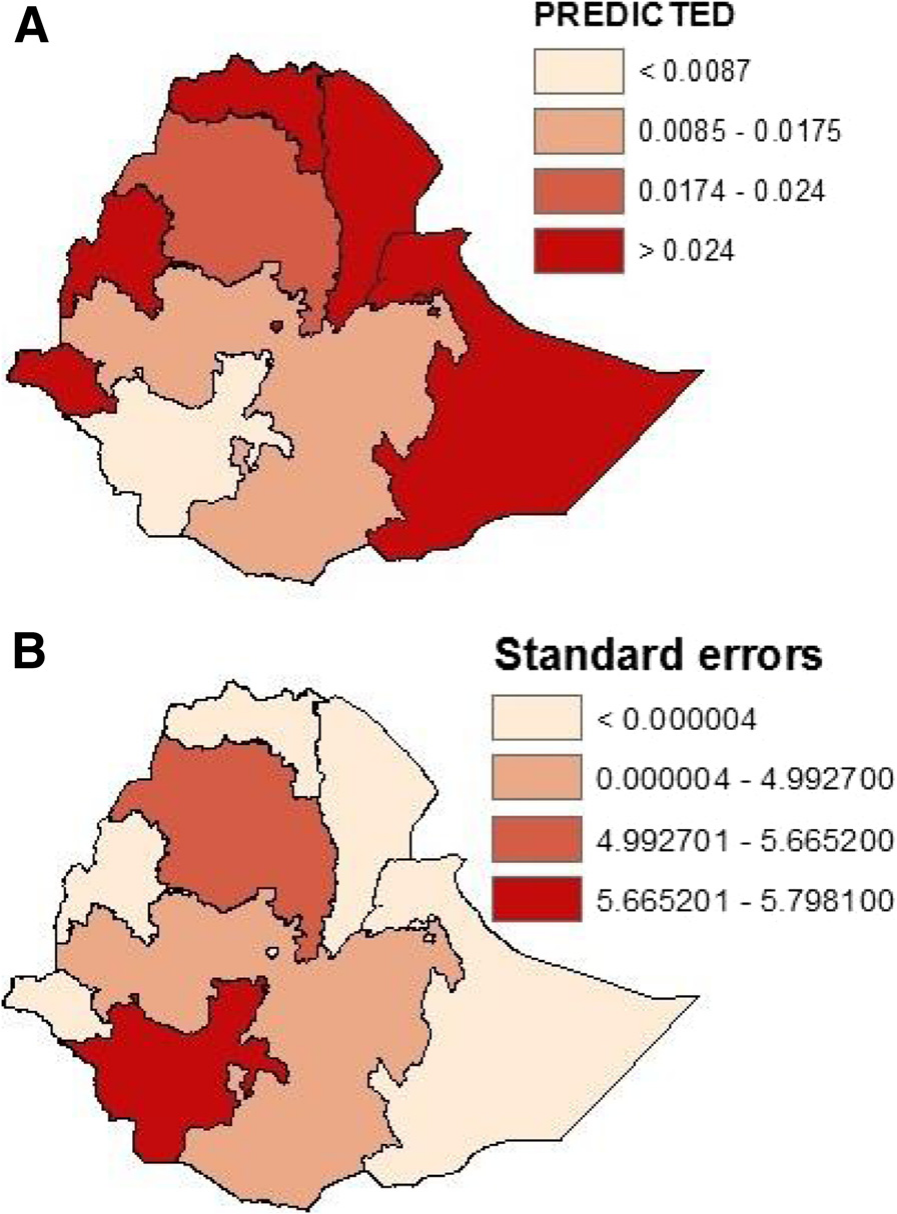
**Risk map of child mortality A: Predictive risk map of child mortality, B: Standard errors associated with the risk map of child mortality.**

Using socio-economic, demographic and geographic indicator variables only, child mortality risk map for Ethiopia was generated (Figure 3A). The risk map shows that, in general, Tigray, Afar, Somali and Benshangul-Gumuz regions had the highest risk followed by Amhara region. In Oromia region, the risk was lower compared to other regions. Addia Ababa, Dire Dawa and Harari regions showed lower risk which could be due their being better health facilities in these regions. Figure 3B shows a map of standard errors, indicating that the highest errors are found in the SNNP regions followed by Amhara and Oromiya regions, compared to the rest of the country.

## Discussion

We analyzed the data from the 2011 Ethiopian Demographic and Health Survey (EDHS) in Ethiopia to estimate the changes in risk factors of child mortality in relation to ongoing interventions by the government of Ethiopia. The 2011 EDHS is the follow-up survey to the 2000 and 2005 EDHS surveys. The 2011 survey provided data which could be used to update estimates of basic demographics and health indicators. The data includes information on background characteristics (age, education, media exposure, etc.), birth history and childhood mortality, knowledge and use of family planning methods, fertility preferences, antenatal, delivery, and postnatal care, breastfeeding and infant feeding practices, vaccinations and childhood illnesses, marriage and sexual activity, women’s work and husband’s background characteristics, awareness and behavior regarding AIDS and other sexually transmitted infections (STIs), adult mortality, including maternal mortality, knowledge of tuberculosis and some geographical variables.

Various researchers have studied the determinants of child mortality in Ethiopia and their findings support the determinants obtained in our study [4,5,32,33]. However, a further statistical analysis is required to identify the factors that determine child mortality. For this reason the structured additive regression models which includes the random effect has been used in this study. A model which allows possible nonlinear relationships between continuous covariates and the response is very important. This model is an adaptation of standard GLMs. In addition to nonlinear structure, taking into consideration the inherent spatial correlation in the data, leads to more accurate estimates of the risk factors of child mortality. Models fitted without taking into account the spatial structure are found to be less adequate when compared with spatial models. Therefore, the results of this study are expected to lead to better estimates of the risk.

The analysis showed that the risk of child mortality increases as the family size approaches to seven and keeps decreasing. But, lower child mortality risk was observed for families with fewer members. Moreover, the highest child mortality was seen in households with more under-five children in the house. Other studies conducted to identify the relationship between child mortality and family size have found that there is clear association between family size and the number of children who have died [34,35]. On the other hand, the relationship between age at first birth and child mortality shows that the risk increases up to age eighteen but starts to fall. Similarly, the relationship between at age first sex shows that the risk of under-five child mortality goes up to age 20 then starts to decrease. These findings from previous studies [32,33] corroborates the results of our study. The risk map produced showed lowest child mortality risk in the Addis Ababa, Dire Dawa and Harari regions compared to the other regions of Ethiopia. The highest risk is observed Tgray, Afar and Amhara regions.

In general, the analysis and risk maps produced in this study indicate that the various risk factors especially geographical ones, such as location of residence (urban or rural) and region remain significant. In addition, other socio-economic factors such as distance to fetch water, source of drinking water, cooking fuel, toilet Facility, floor material, roof material, wall material and smoking have significant effects.

The 2011 EDHS acts as a continuation survey on which subsequent surveys will be built. It is crucial to monitor trends in child mortality risk and to continually explore the complex relationships between child mortality risk and socio-economic, demographic and geographic factors. This will be possible since each round of the EDHS will cover the same locations thus making it possible to monitor under-five child mortality risk over a long period of time. Furthermore, effective control measures of under-five mortality at household level in Ethiopia should start with proper mapping of the risk. This will help to understanding the distribution of under-five mortality so that resources can be prudently allocated to deal with the problem.

## Conclusion

Our findings support the notion that childhood mortality is an increasing public health issue in the country with spatial variation across different regions. The results of this study suggested that there are complex social, demographic and geographic processes operating in under-five mortality. This result can be more clearly understood using the appropriate statistical models. Therefore, this study provides an empirical child mortality risk map that can be used for intervention by the government. Identifying areas that are likely to have higher child mortality risks helps to take measures in those areas that require special attention. The results of this study can lead to a better understanding of the spatial distribution of child mortality and together with expert opinion, which is widely used in the absence of empirically produced maps; they can be useful to avoid the risk.

One of the limitations in the present study is that it is not possible to produce risk maps at the lowest administrative units as the DHS data is not sampled by clustering the region/country by the lowest administrative units, called *Wereda*. The other limitation is the non-exhaustiveness of the factors which might be relevant to child mortality. But, the variables are not included in the DHS. The advantage in the use of DHS data is the issue of data quality. The goodness of the DHS data is the quality. The survey uses complex questionnaires which allows for inconsistent responses to be detected very easily. The novelty of this study is identifying the key risk factors for child mortality in Ethiopia the use of the recently developed Structured Additive Regression Models which provides valid and realistic statistical inferences.

Ethiopian Demographic and Health Survey (EDHS) is conducted every five years. For further analysis, the available EDHS surveys could be merged together and the time effect (year of the survey) will be included in the analysis. Therefore, for this study, we propose a general class of structured additive regression model for categorical response, allowing flexible semiparametric predictors, i.e., nonlinear effects of continuous covariates, time trends and interaction between time variable and continuous covariates; and between continuous covariates. This model will be implemented to see the effect of socio-economic, demographic and geographic factors through years. This process will be useful to see the trends of child mortality through years.
